# Prevalence and Predisposing Factors for Recurrence after Hallux Valgus Surgery: A Systematic Review and Meta-Analysis

**DOI:** 10.3390/jcm10245753

**Published:** 2021-12-09

**Authors:** Yasmin Ezzatvar, Laura López-Bueno, Laura Fuentes-Aparicio, Lirios Dueñas

**Affiliations:** 1 Department of Nursing, University of Valencia, 46010 Valencia, Spain; yasmin.ezzatvar@uv.es; 2 Department of Physiotherapy, University of Valencia, 46010 Valencia, Spain; 3 Physiotherapy in Motion, Multi Speciality Research Group (PTinMOTION), Department of Physiotherapy, University of Valencia, 46010 Valencia, Spain; laura.fuentes@uv.es (L.F.-A.); lirios.duenas@uv.es (L.D.)

**Keywords:** recurrence, scarf osteotomy, akin osteotomy, hallux valgus angle, intermetatarsal angle, bunion, foot

## Abstract

Recurrence is a frequent and undesirable outcome after hallux valgus (HV) surgery. However, the prevalence of HV recurrence and the pre- and postoperatory factors associated with it have not been adequately studied. This study aimed to quantify the prevalence rate of HV recurrence and to analyze its predisposing factors. MEDLINE and EMBASE databases were systematically searched for observational studies including individuals undergoing HV surgical correction. The random-effects restricted maximum likelihood model was used to estimate the pooled effect size (correlation coefficient (r)). Twenty-three studies were included, yielding a total of 2914 individuals. Pooled prevalence of HV recurrence was 24.86% (95% confidence interval (CI), 19.15 to 30.57, I^2^ = 91.92%, *p* = 0.00). Preoperative HV angle (HVA) (r = 0.29; 95% CI, 0.14 to 0.43) and preoperative intermetatarsal angle (IMA) (r = 0.13; 95% CI, 0.00 to 0.27) showed a moderate positive relationship with recurrence. Postoperative HVA (r = 0.57; 95% CI, 0.21 to 0.94) and sesamoid position (r = 0.46; 95% CI, 0.31 to 0.60) showed strong relationships with recurrence. In conclusion, preoperative HVA, IMA, and postoperative HVA and sesamoid position are significant risk factors for HV recurrence, and the association of these factors with recurrence is affected by age.

## 1. Introduction

Hallux valgus (HV) is a common foot deformity, which affects nearly 23% of adults aged 18–65 years, and is more common in women and with increasing age [1]. Mild cases are generally treated with conservative methods, but patients with severe and painful deformity are usually referred for surgical correction. However, the optimum procedure to correct HV remains to be defined, and more than 130 different surgical techniques are described in the literature [2]. Unfortunately, when HV treatment is inadequately prescribed or executed, it frequently results in low levels of patient satisfaction, raising issues of quality of life and functionality outcomes, especially after HV surgery [3,4].

Recurrent HV is a frequent postoperative complication, with rates as high as 73% [5], although the mechanisms that underpin recurrence after HV surgical correction are not fully understood. A previous review showed that recurrence has a multifactorial etiology with a combination of factors including the anatomical predisposition of the patient, surgical factors, medical comorbidities and compliance with postsurgical instructions following surgery [6]. In the literature, recurrence rates vary significantly across studies, most likely due to different definitions of recurrence, and also due to the postoperative time frame reported for a recurrence [7,8,9]. Furthermore, causal factors leading to recurrent HV remain speculative, and no rigorous meta-analysis of studies quantifying recurrence rates following HV surgery and predisposing risk factors has been published.

Within this context, it is incumbent on foot and ankle specialists to identify potential risk factors for HV recurrence not only to prevent a considerable proportion of recurrent HV, but also to improve treatment outcomes. To address these important evidence gaps, the present study sought to quantify the general prevalence of HV recurrence and its determinants in patients who underwent HV surgery by performing a meta-analysis of observational studies.

## 2. Materials and Methods

The present systematic review and meta-analysis was conducted following the Preferred Reporting Items for Systematic Reviews and Meta-Analyses (PRISMA) guidelines. The study was submitted to the International Prospective Register of Systematic Reviews (PROSPERO) (registration number: CRD42021286981). The whole process from literature selection to data extraction was performed independently by 2 researchers (YE and LD). Any disagreements were resolved through consensus with a third researcher (LLB).

### 2.1. Selection Criteria

To be eligible for inclusion in the meta-analysis, studies needed to meet the following criteria (using PECOS criteria): (i) participants: individuals who underwent surgery for HV; (ii) exposure: pre/post-surgical HV assessed through standardized weight-bearing radiographs; (iii) comparisons: recurrent vs. non-recurrent HV; (iv) outcomes: pre- and postoperative HV angle (HVA), intermetatarsal angle (IMA), distal metatarsal articular angle (DMAA), sesamoid position, joint congruence, shape of the first metatarsal head, foot deformities, age; (v) study design: prospective cohort studies. Studies were excluded if they: (a) did not report data regarding the variables of interest; (b) measured recurrence through non-radiographic methods; (c) included patients who did not undergo a primary HV surgery correction; or (d) reported insufficient information for calculating correlation coefficients (r) and 95% confidence intervals (95% CIs). The first and second reviewers (YE and LD) assessed the full-text articles for eligibility. If a single study assessed different risk factors (e.g., HVA, IMA, age), all effect sizes were extracted.

### 2.2. Search Strategy

Two authors (YE and LD) methodically searched MEDLINE and EMBASE electronic databases for articles, from inception to November 2021. We used the terms vallux valgus, hallux abductus varus, recurrence, recidive, HV surgery, osteotomy, scarf, Akin, chevron, Ludloff, Lapidus, bunion, bunionectomy, radiographic assessment, hallux valgus angle, intermetatarsal angle, distal metatarsal articular angle, sesamoid position or subluxation (Supplementary Material File S1). Searching was restricted to peer-reviewed articles published in English or Spanish language, or at least to published articles in which the abstract and the variables of interest were described in English or Spanish language.

### 2.3. Data Collection Process and Data Items

The extracted data from the studies that met the inclusion and exclusion criteria included the following information: (i) study characteristics (the first author’s name, publication year, enrolment year, study location, sample size, study design); (ii) participants’ information (sex and age); (iii) measurements details (radiograph procedure and angle measurement); and (iv) analysis and study results (adjusted variables, outcome of interest and main results). We contacted the corresponding authors of the studies via e-mail to request any effect sizes that were missing from the original published papers.

### 2.4. Risk of Bias in Individual Studies

The Quality Assessment Tool for Observational Cohort and Cross-sectional Studies was applied to assess the risk of bias [10]. The checklist comprised 14 items for longitudinal research. Each item of methodological quality was classified as “yes”, “no” or “not reported”.

### 2.5. Summary Measures

The main effect size for our study was the correlation coefficient (r). Effect sizes obtained from the studies were standardized and unstandardized regression coefficients (β and beta, respectively), standardized mean differences (Cohen’s d), and odds ratios (ORs). All these estimates were converted to correlations by using their corresponding formulas [11,12,13]. In the case of studies reporting adjusted and unadjusted effect sizes, the adjusted effect sizes were chosen. The meta-analysis software was configured to produce pooled r values with 95% CIs using the random-effects restricted maximum likelihood model, from the correlation coefficients and the standard error or sample size from each study. The effect size for r was categorized as small (≤0.10), moderate (0.10–0.37) or large (≥0.37) [14]. All analyses were performed using the admetan routine 16 within version 13.1 of STATA (STATA Corp., College Station, TX, USA). A *p*-value of <0.05 was considered the threshold for statistical significance. Additionally, the prevalence of recurrence after HV surgery across studies was pooled by applying a random-effects model that displayed the results as forest plots using the DerSimonian and Laird method (metaprop procedure [15]). The Clopper–Pearson method was used to establish confidence intervals for prevalence from the selected individual studies [16] and a Freeman–Tukey transformation was used to normalize the results before calculating the pooled prevalence [17].

### 2.6. Synthesis of Results

Heterogeneity across studies was analyzed using the total variance (Q), the degrees of freedom (df) and the inconsistency index (I^2^) [18] for each meta-analysis, considering I^2^ values of <25%, 25–75%, and ≥75% as small, moderate and high heterogeneity, respectively [19]. Sensitivity analyses were conducted to ascertain whether any individual study with extreme results had an unjustified effect on the overall results.

### 2.7. Risk of Bias across Studies

The risk of bias across studies was analyzed using the Luis Furuya-Kanamori (LFK) index and the Doi plot, respectively. Both tests have been shown to be more robust than the traditional funnel plot and Egger’s regression intercept test [20]. Values of −1, between −1 and −2, and >−2, are considered to represent no, minor, and major asymmetry, respectively [20].

## 3. Results

### 3.1. Study Selection

**Figure 1:**
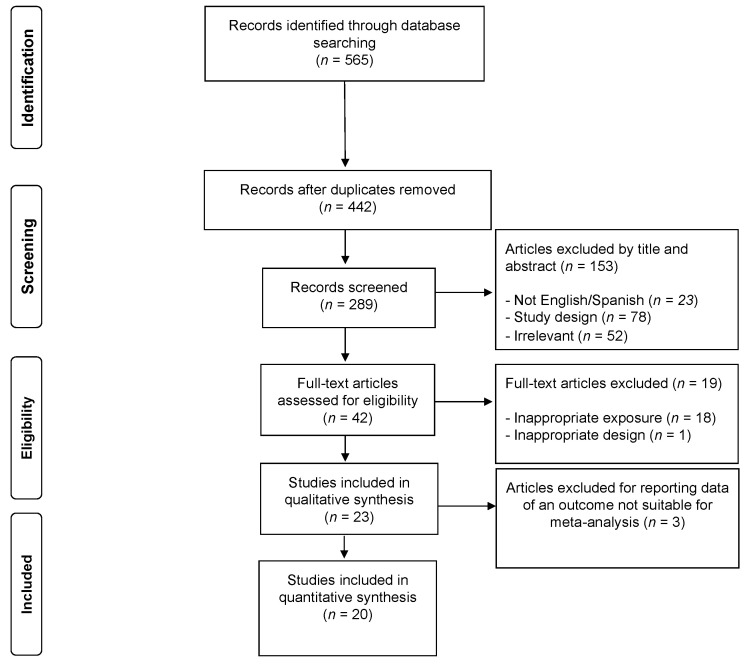
PRISMA flow diagram of literature search and study selection.

In total, 23 studies were included in the systematic review, although only 20 met the criteria for inclusion in the meta-analysis. The PRISMA flow diagram illustrating the number of studies excluded at each stage of the systematic review and meta-analysis is shown in Figure 1.

### 3.2. Study Characteristics

A total of 23 prospective cohort studies reported the associations between pre- and postoperative factors and recurrence following HV surgery, and details of these are listed in Table 1. The included studies involved a total sample of 2914 individuals with ages ranging from 14 to 84 years (mean age = 49.7 years). Sample sizes across studies ranged from 17 [21] to 587 [22] participants. Five studies included only females [7,23,24,25,26], and although the remaining 18 studies included a mixed sample, females comprised the majority of the studied population. Follow-up duration ranged from 1.5 [27] to 124 months [28] (mean follow-up length = 42.7 months).

Regarding the study location, eleven studies were conducted in Asian countries, including Korea [29,30,31], Japan [7,24,25], Singapore [27,32], China [33], Turkey [9] and Hong Kong [26], three in the USA [8,21,22] and six in European countries, including Finland [5], Austria [28,34], Germany and Switzerland [35], Greece [36] and the Netherlands [37]. In the remaining three studies, study location was not reported [23,38,39].

Surgical procedures varied across studies, including proximal metatarsal osteotomy [7,24,25], scarf osteotomy [9,27,28,32,35,36,38,39], chevron osteotomy [5,23], Ludloff osteotomy [29], syndesmosis procedure [26] or a combination thereof [8,21,22,31,33,34,37]. Recurrence rates ranged from 9% [31] to 73% [5].

### 3.3. Measurements

Regarding the parameters used, studies reported data on IMA [5,8,9,21,23,26,27,28,31,34,35,36,37,38,39], HVA [8,23,25,28,29,30,31,33,38,39], DMAA [30,31,38], sesamoid position [8,24,27,38], joint congruence [9,38], the shape of the lateral edge of the first metatarsal head [7] or the presence of a foot deformity [22,39] through radiographs. Other parameters were generalized ligamentous laxity [23], surgical procedure [29] and age [32].

Finally, eight of the twenty-three studies defined recurrence as HVA ≥ 20° [7,24,25,28,29,30,32,33], six studies considered recurrence as HVA > 20° [22,23,31,34,35,38], three studies defined it as HVA > 15° [5,9,39], one study considered recurrence when the HVA increased ≥3° during the follow-up [8], and another study considered recurrence when an increase of ≥5° postoperatively was observed [21]. The remaining studies did not report a definition for recurrence (Table 1).

### 3.4. Risk of Bias within Studies

All 23 studies met at least four criteria and were considered to have moderate methodological quality. The average score was 7.74/14.0 (Supplementary Material Table S1).

### 3.5. Synthesis of Results

**Figure 2:**
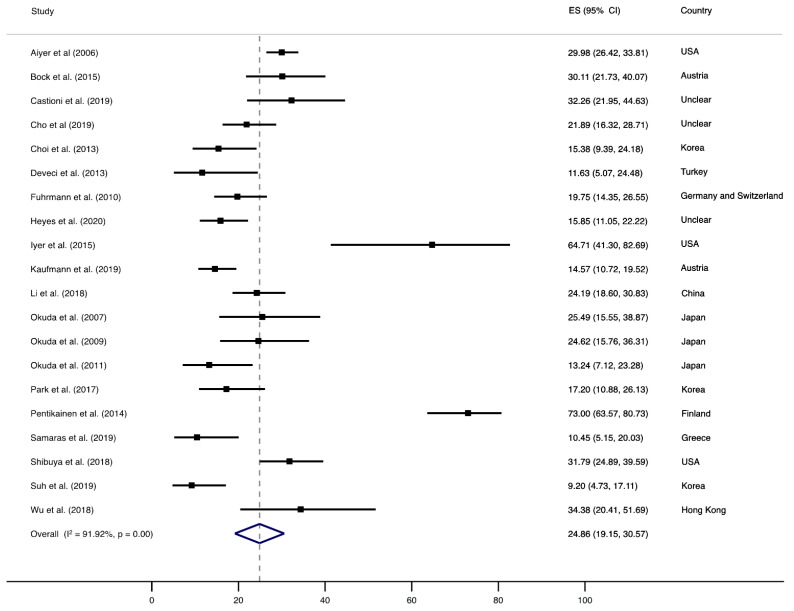
Forest plot showing the pooled prevalence of HV. Squares represent the pooled prevalence for each study, and the diamond represents the overall pooled prevalence.

**Figure 3:**
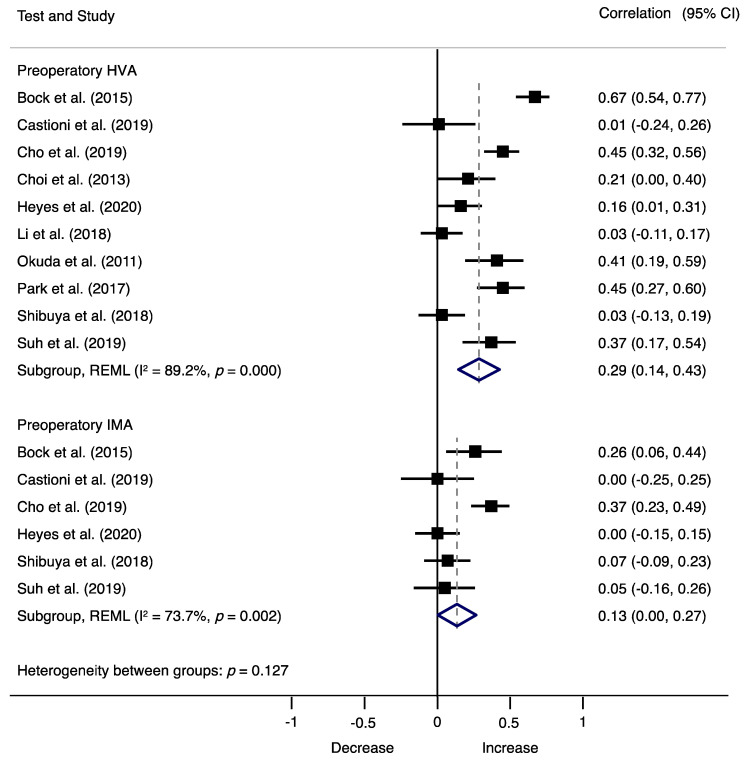
Forest plot showing the correlation of HV recurrence and preoperatory factors for each study. Squares represent pooled effect size for each subgroup analysis, and the diamond represents the overall pooled effect size. HVA, hallux valgus angle; IMA, intermetarsal angle.

**Figure 4:**
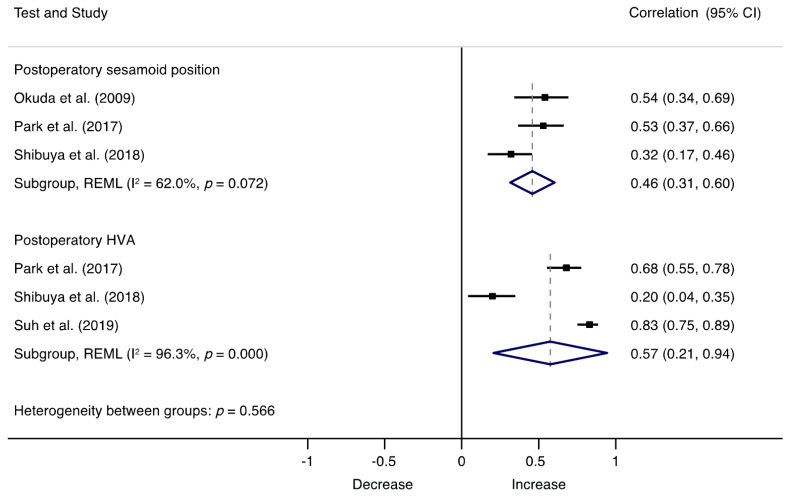
Forest plot showing the correlation of HV recurrence and postoperatory factors for each study. Squares represent pooled effect size for each subgroup analysis, and the diamond represents the overall pooled effect size. HVA, hallux valgus angle.

Figure 2, Figure 3 and Figure 4 show the synthesis of results. Pooled prevalence of recurrence after HV surgery was 24.86% (95% confidence interval (CI), 19.15 to 30.57, I^2^ = 91.92%, *p* = 0.00), as shown in Figure 2.

Associations between preoperative factors and recurrence are shown in Figure 3, where preoperative HVA (r = 0.29; 95% CI, 0.14 to 0.43, I^2^ = 89.2%, Q = 83.58) and preoperative IMA (r = 0.13; 95% CI, 0.00 to 0.27, I^2^ = 73.7%; Q = 19.02) showed a moderate positive relationship with recurrence, respectively, and no asymmetry suggestive of small-study effects (LFK index = −0.99) (Supplementary Material Figure S1). Sensitivity analyses showed no changes in the findings after removing one study at a time. Meta-regression analyses showed no significant effects of length of follow-up (β = 0.02 95% CI, −0.000 to 0.005; *p* = 0.076) (Supplementary Material Figure S2), but significant effects were detected with age (β = −0.02 95% CI, −0.036 to 0.000; *p* = 0.046) (Supplementary Material Figure S3).

The associations between postoperatory factors and recurrence are illustrated in Figure 4, where postoperatory HVA (r = 0.57; 95% CI, 0.21 to 0.94, I^2^ = 96.3%, Q = 54.74) and sesamoid position (r = 0.46; 95% CI, 0.31 to 0.60, I^2^ = 62.0%, Q = 5.27) showed strong relationships with recurrence, with major asymmetry suggestive of small-study effects (LFK index = −2.95) (Supplementary Material Figure S1). Sensitivity analyses indicated that results remained consistent across all deletions. Meta-regression analyses showed no significant effects of length of follow-up (β = 0.08 95% CI, −0.012 to 0.299; *p* = 0.332) (Supplementary Material Figure S4), but significant effects were detected with age (β = −0.43 95% CI, −0.064 to −0.022; *p* = 0.004) (Supplementary Material Figure S5).

## 4. Discussion

The present study aimed to examine the recurrence rate of HV derived from the available scientific literature and to disentangle the predisposing factors associated with recurrence following HV surgery. Findings suggest that HV recurrence occurs in approximately one-quarter of patients undergoing corrective surgery (24.86%), and it is associated with many potential factors, including preoperative HVA and IMA, and postoperative HVA and sesamoid position. These findings highlight the need to identify adequate strategies aimed to reduce or prevent recurrence following HV surgery.

Increased preoperative HVA and IMA were found to be significant risk factors for HV recurrence, regardless of the surgical technique used, ruling out a potential relationship between a specific surgical technique and recurrence. For this reason, a possible explanation for these associations may be related to the selection of the surgical procedure. Surgery success relies, at least partly, on the ability of the operator to recognize the underlying causes of HV in each patient, and this decision partially depends on the level of deformity and extent of correction required. In this context, the IMA has been traditionally considered to guide treatment, and different approaches have been indicated according to this angle: distal osteotomies for mild to moderate HV deformities, and when more correction is needed, more proximal first metatarsal osteotomies or first tarsometatarsal fusions are recommended [40]. However, the existing body of research on HV currently considers its multiplanar structure and the rotational deformity of the first ray [41,42,43], and it has been suggested that the ability to attain a satisfactory correction may be influenced by ‘derotating’ the first metatarsal bone [42]. Because surgical options are generally aimed to treat the metatarsus varus deviation, but not any rotation [44], it could be argued that the undercorrection of a pronated metatarsal may account, at least partly, for some cases of recurrence in patients with increased HVA or IMA.

Furthermore, the possible interference of other factors related to surgery, such as the execution of the procedure, which depends largely on the surgical expertise of the operator, cannot be ruled out. For instance, some metatarsal osteotomies are more technically demanding than others, have a steeper learning curve, and need special attention to the 3D changes when performing it [45]. Although we could not ascertain the surgical expertise of the surgeons participating in the studies and, therefore, it was not possible to measure the influence of any “learning curve”, it could conceivably be hypothesized that a lack of experience and/or a poor execution of the procedure could have contributed to our results.

Regarding postoperative factors, we found that the immediate postoperative HVA was strongly associated with recurrence. The immediate postoperative HVA reflects the correction achieved immediately after the surgery; an increased HVA may reflect an undercorrection of the surgery, and therefore, a recurrence of the deformity according to the definition of recurrence used in most studies (that is, a HVA higher than 15° or 20°, depending on the study). Likewise, a tibial sesamoid position greater than 4 was strongly correlated with recurrence. This position represents the location of the internal sesamoid relative to the middle line of the metatarsophalangeal joint, where 1 is considered a normal position and 7 denotes complete luxation [46]. This malposition of the internal sesamoid might be the result of the HV rotational forces that push the sesamoid complex after the metatarsal through several ligamentous connections between the first metatarsal bone, the hallux, and the sesamoids [44]. Because a complete correction of HV entails the correction of the rotation component of the deformity, it could be assumed that a tibial sesamoid position greater than 4 after surgery may imply that total correction of the deformity was not achieved, this being a possible explanation for its relationship with recurrence. It has been suggested that the use of computerized axial tomography imaging in evaluations of HV may be useful for surgeons when they make operative choices to treat this condition, considering that up to 87% of patients with HV deformities have a pronated first metatarsal, with or without sesamoid subluxation [47].

Very little was found on whether joint congruence is associated with recurrence because not enough information was provided to assess this association. However, previous reports have shown significant associations between first metatarsophalangeal joint incongruity on preoperative radiographs and recurrent HV following a scarf osteotomy [9,38]. Although further research is needed to confirm this association, the presence of incongruence in the first metatarsophalangeal joint should be carefully considered when planning surgery for HV, as it could potentially increase the risk of recurrence. Moreover, available data on compliance with postoperative care instructions were scarce, so it was not possible to quantify its association with recurrence. This factor was only measured in the study of Castioni et al., who reported that patients with poor compliance to postoperative care instructions had higher risk of developing a larger postoperative HVA and, therefore, HV recurrence [38]. Additionally, meta-regression analyses indicated that the duration of follow-up did not moderate the recurrence correlation coefficients. However, age significantly increased these associations, being a potential factor to consider when planning a surgical intervention.

To date, evidence to accurately evaluate the treatment efficacy of the different types of surgical procedures for HV in terms of recurrence have been poorly conducted and reported. In this context, the optimum procedure to be performed remains to be defined, albeit the proper identification of the causes of HV, the right choice of the technique, and the surgical expertise of the surgeons might be determinants for reducing the recurrence after HV surgery. Further studies should be undertaken to investigate the associations between other potential risk factors and recurrence using standardized definitions for recurrent HV.

### Limitations and Strengths

Our findings should be considered in light of several limitations in the available literature concerning HV recurrence. First, correlations denote an association between the past and the present but are unable to predict the future and do not determine the cause or the effect of any process. Second, our meta-analysis identified considerable heterogeneity across studies, which could have biased our results. Third, our results were limited by the lack of a uniformly accepted definition of HV recurrence, reflecting an inconsistency in the use of a standardized definition of recurrence, and thereby the use of diverse thresholds to detect patients with recurrence. Moreover, although we report a pooled recurrence rate of 24.86%, only 20 of 23 studies including 2535 patients reported recurrence, and since studies used different definitions of HV recurrence, we cannot confirm that our pooled recurrence rate reflects the true HV recurrence. Further prospective studies using standardized definitions of recurrence after HV surgery are therefore needed. Likewise, our results are limited by the scarcity of high-quality studies and by the wide variety in risk factor definitions, which restricted our capacity to analyze all available data in the statistical analysis. On the other hand, this study also has strengths that must be acknowledged. This is the first study that quantifies the associations between pre- and postoperatory factors with recurrence after HV surgery and provides an estimate of the recurrence rate, laying the groundwork for future research in the field.

## 5. Conclusions

Our meta-analysis demonstrates that the risk of recurrence after HV surgery is 24.86%, and preoperative HVA and IMA, and postoperative HVA and sesamoid position are significant risk factors for HV recurrence. Although the optimum procedure remains to be defined, our results may help foot and ankle specialists inform patients, choose the appropriate surgical technique according to the causes of HV, and design and plan surgical intervention trials.

## Figures and Tables

**Table 1:** Characteristics of the studies.

Study	Country	*n* (Females)	Mean Age (Range)	Follow-Up (Months)	Recurrence Definition	Recurrence Rate	Surgical Procedure
Aiyer et al., (2006)	USA	587	NR	12	HVA > 20°	30%	Distal first metatarsal osteotomy (chevron), proximal first metatarsal ostetomy (scarf or Ludloff) or a Lapidus procedure
Bock et al., (2015)	Austria	93 (87)	50 (21–78)	124	HVA ≥ 20°	30%	Scarf osteotomy
Castioni et al., (2019)	Unclear	62 (56)	57	38	HVA > 20°	32.8%	Scarf osteotomy
Cho et al., (2019)	Unclear	169 (169)	38 (18–58)	46.3	HVA > 20.0°	21.7% and 17.1%	Proximal chevron osteotomy
Choi et al., (2013)	Korea	91 (89)	52 (22–74)	26	HVA ≥ 20.0°	15.50%	Ludloff osteotomy combined with additional procedures
Deenik et al., (2008)	The Netherlands	136 (118)	43.5	28.8	NR	12 cases had severe recurrences	Bunionectomy, osteotomy, lateralization of the distal fragment, lateral release and medial capsulorrhaphy
Deveci et al., (2013)	Turkey	43 (36)	47.7 (21–65)	26.2	HVA > 15.0°	11.62%	Scarf osteotomy
Fuhrmann et al., (2010)	Germany and Switzerland	162 (156)	53.8 (17–77)	44.9	HVA > 20.0°	19.70%	Scarf osteotomy
Goh et al., (2021)	Singapore	193 (179)	53.9 (28–82)	110.4	HVA ≥ 20.0°	NR	Scarf osteotomy and additional procedures (Akin osteotomy and Weil osteotomy)
Heyes et al., (2020)	Unclear	164 (154)	52	6	HVA > 15.0°	16%	Scarf osteotomy
Iyer et al., (2015)	USA	17 (14)	47.7 (14–71)	28.8	Increased > 5° HVA postoperatively	64.70%	Proximal medial opening wedge osteotomy, and associated procedures (Akin osteotomy, second hammertoe correction and medial sesamoidectomy for osteonecrosis)
Kaufmann et al., (2019)	Austria	247(224)	52.1	45.4	HVA ≥ 20.0°	14.70%	Scarf osteotomy (group S, *n* = 184) and additionally Akin osteotomy (group SA, *n* = 63 patients)
Li et al., (2018)	China	186	56.5 (17–84)	83.7	HVA ≥ 20.0°	24.20%	Chevron osteotomy combined with distal soft tissue procedure, Akin osteotomy, Weil osteotomy
Okuda et al., (2007)	Japan	51 (51)	53	48	HVA ≥ 20.0°	25%	Proximal metatarsal osteotomy
Okuda et al., (2009)	Japan	65 (65)	51	45	HVA ≥ 20.0°	25%	Proximal metatarsal osteotomy
Okuda et al., (2011)	Japan	68 (68)	53	33	HVA ≥ 20.0°	13.90%	Proximal metatarsal osteotomy
Park et al., (2017)	Korea	93 (91)	51	27.5	HVA ≥ 20.0°	17.10%	Chevron osteotomy combined with distal soft tissue procedure
Pentikainen et al., (2014)	Finland	100 (92)	39	94.8	HVA > 15°	73%	Chevron osteotomy
Samaras et al., (2019)	Greece	67 (65)	53.6	24	NR	10.44%	Scarf osteotomy
Seng et al., (2015)	Singapore	71	48.7	1.5	NR	NR	Scarf osteotomy
Shibuya et al., (2018)	USA	151 (140)	57	6	HVA of ≥3° after ≥6 months postoperatively	32%	Distal metatarsal osteotomy, modified Lapidus procedure and proximal/midshaft/double metatarsal osteotomies
Suh et al., (2019)	Korea	87	44	20.6	HVA > 20.0	9%	Scarf and Akin osteotomy
Wu et al., (2018)	Hong Kong	32 (32)	39 (14 to 63)	63.2	MPA > 20°	35%	Syndesmosis procedure

Abbreviations: HVA, hallux valgus angle; IMA, intermetatarsal angle; MA, metatarsus adductus; MPA, metatarsophalangeal angle; NR, not reported.

## Data Availability

Data are available upon reasonable request.

## Supplementary Materials

The following are available online at https://www.mdpi.com/article/10.3390/jcm10245753/s1: Supplementary Material File S1: Electronic search strategy; Supplementary Material Table S1: Results of the Items of Quality Assessment Tool for Observational Cohort and Cross-sectional studies; Supplementary Material Figure S1: Small-study effects and publication bias represented by Doi plots and the Luis Furuya-Kanamori (LFK) index; Supplementary Material Figure S2: Association between correlations on recurrence according to preoperatory factors and follow-up (months); Supplementary Material Figure S3: Association between correlations on recurrence according to preoperatory factors and age (years); Supplementary Material Figure S4: Association between correlations on recurrence according to postoperatory factors and follow-up (months); Supplementary Material Figure S5: Association between correlations on recurrence according to postoperatory factors and age (years).
