# Supplementary material for: Prevalence and Predisposing Factors for Recurrence after Hallux Valgus Surgery: A Systematic Review and Meta-Analysis
Source: J Clin Med. 2021 Dec 9;10(24):5753. doi: 10.3390/jcm10245753 (PMC8708542; doi:10.3390/jcm10245753)

Supplementary Material

Prevalence and predisposing factors for recurrence after Hallux Valgus surgery.

A systematic review and meta-analysis.

Supplementary File S1. Electronic search strategy.

Supplementary Table S1. Results of the Items of Quality Assessment Tool for Observational Cohort and Cross-sectional studies.

Supplementary Figure S1. Small-study effects and publication bias represented by Doi plots and the Luis Furuya-Kanamori (LFK) index.

Supplementary Figure S2. Association between correlations on recurrence according to preoperatory factors and follow-up (months).

Supplementary Figure S3. Association between correlations on recurrence according to preoperatory factors and age (years).

Supplementary Figure S4. Association between correlations on recurrence according to postoperatory factors and follow-up (months).

Supplementary Figure S5. Association between correlations on recurrence according to postoperatory factors and age (years).

This supplementary material has been provided by the authors to give readers additional information about their work.

Supplementary File S1. Electronic search strategy.

MEDLINE

((("Hallux Valgus "[Title/Abstract] OR "Hallux abductus varus"[Title/Abstract]) AND ("recurrence"[Title/Abstract] OR "recidive"[Title/Abstract]) AND "surgery"[Title/Abstract] OR "osteotomy"[Title/Abstract] OR "Scarf"[Title/Abstract] OR "Akin"[Title/Abstract] OR Chevron[Title/Abstract] OR "Ludloff"[Title/Abstract] OR "Lapidus"[Title/Abstract] OR "bunionectomy"[Title/Abstract] OR "bunion"[Title/Abstract])) AND ("radiographic assessment"[Title/Abstract] OR "intermetatarsal angle"[Title/Abstract] OR "distal metatarsal articular angle"[Title/Abstract])) AND (longitudinal[Title/Abstract] OR prospective[Title/Abstract] OR "follow-up"[Title/Abstract])

(417 results)

Supplementary Table S1. Results of the Items of Quality Assessment Tool for Observational Cohort and Cross-sectional studies.

Study	1	2	3	4	5	6	7	8	9	10	11	12	13	14	Total score
Aiyer et al (2006)	✓	✓	✓	✓	✓	×	×	✓	✓	×	✓	NR	NR	✓	9
Bock et al. (2015)	✓	✓	✓	✓	✓	×	×	✓	✓	×	✓	NR	NR	✓	9
Castioni et al. (2019)	✓	✓	×	✓	×	×	×	✓	✓	×	✓	NR	NR	×	6
Cho et al (2019)	✓	✓	NR	✓	✓	×	×	✓	✓	×	✓	NR	NR	×	7
Choi et al. (2013)	✓	✓	✓	✓	✓	✓	✓	✓	✓	✓	✓	NR	NR	✓	12
Deenik et al. (2008)	✓	✓	✓	✓	✓	×	×	✓	✓	×	✓	NR	NR	✓	9
Deveci et al. (2013)	✓	✓	NR	✓	NR	×	×	✓	✓	×	✓	NR	NR	✓	7
Fuhrmann et al. (2010)	✓	✓	✓	✓	×	×	×	✓	✓	×	✓	NR	NR	NR	7
Goh et al. (2021)	✓	✓	✓	✓	✓	×	×	✓	✓	×	✓	NR	NR	×	8
Heyes et al. (2020)	✓	✓	✓	✓	NR	×	×	✓	✓	×	✓	NR	NR	✓	8
Iyer et al. (2015)	✓	✓	✓	✓	×	×	×	✓	✓	×	✓	NR	NR	×	7
Kaufmann et al. (2019)	✓	✓	✓	✓	✓	×	×	✓	✓	×	✓	NR	NR	×	8
Li et al. (2018)	✓	✓	✓	✓	×	×	×	✓	✓	×	✓	NR	NR	×	7
Okuda et al. (2007)	✓	✓	✓	✓	×	×	×	✓	✓	×	✓	NR	NR	✓	8
Okuda et al. (2009)	✓	✓	✓	NR	×	×	×	✓	✓	×	✓	NR	NR	NR	6
Okuda et al. (2011)	✓	✓	✓	NR	×	×	×	✓	✓	×	✓	NR	NR	✓	7
Park et al. (2017)	✓	✓	✓	NR	NR	×	×	✓	✓	×	✓	NR	NR	✓	7
Pentikainen et al. (2014)	✓	✓	NR	✓	✓	×	×	✓	✓	×	✓	NR	NR	✓	8
Samaras et al. (2019)	✓	✓	✓	✓	✓	×	×	✓	✓	×	✓	NR	NR	✓	9
Seng et al. (2015)	✓	✓	NR	✓	NR	×	×	✓	✓	×	✓	NR	NR	NR	6
Shibuya et al. (2018)	✓	×	✓	✓	✓	×	×	✓	✓	×	✓	NR	NR	NR	7
Suh et al. (2019)	✓	✓	✓	✓	✓	×	×	✓	✓	×	✓	NR	NR	✓	9
Wu et al. (2018)	✓	✓	NR	✓	NR	×	×	✓	✓	×	✓	NR	NR	✓	7

✓ indicates “yes”, × indicates “no”, and NR indicates “not reported”.

Supplementary Figure S1. Small-study effects and publication bias represented by Doi plots and the Luis Furuya-Kanamori (LFK) index.

(Preoperatory factors)

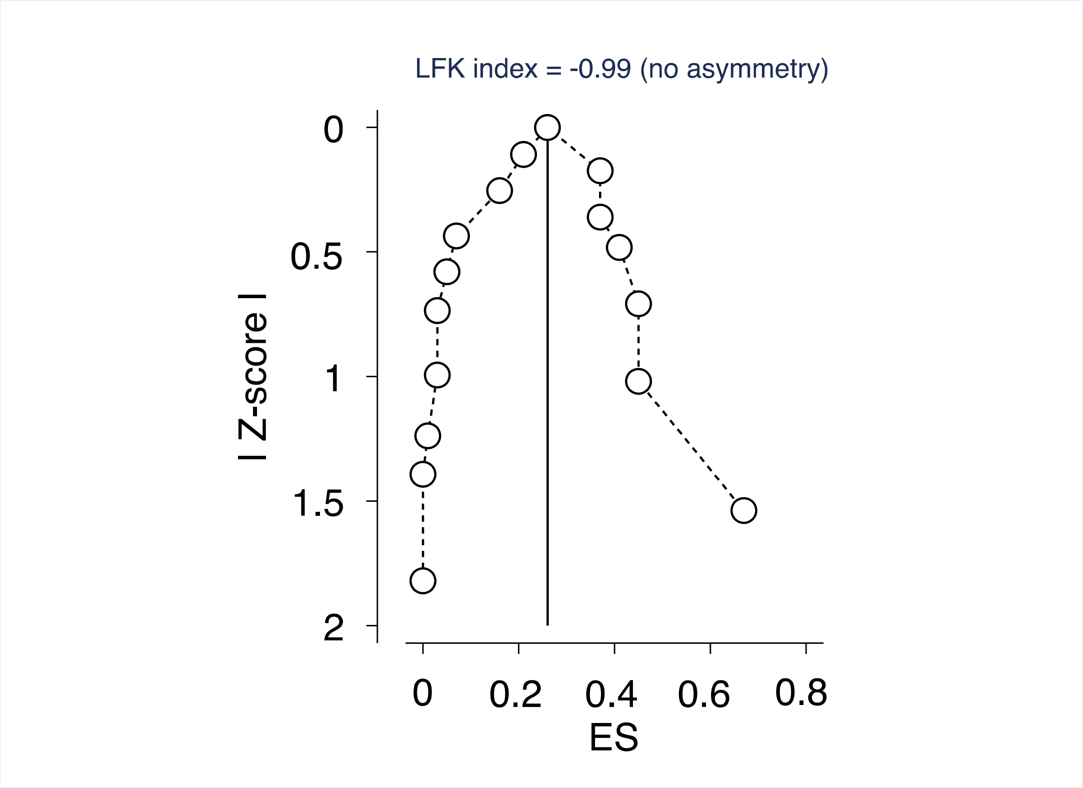

(Postoperatory factors)

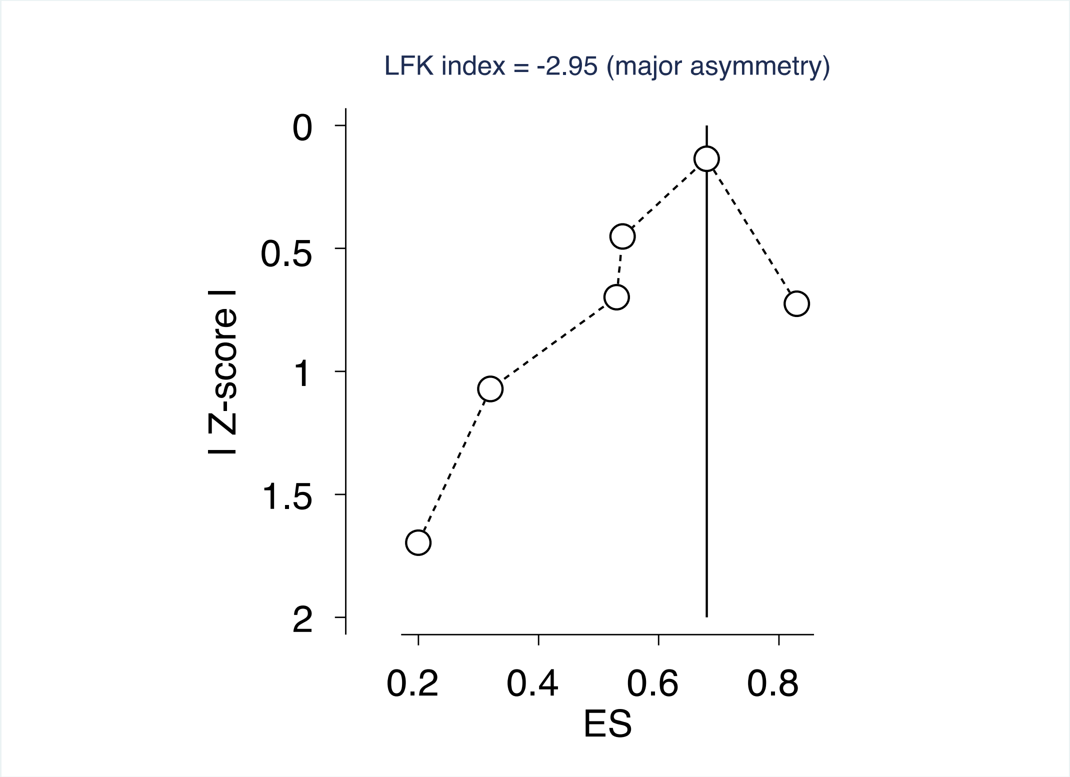

Supplementary Figure S2. Association between correlations on recurrence according to preoperatory factors and follow-up (months).

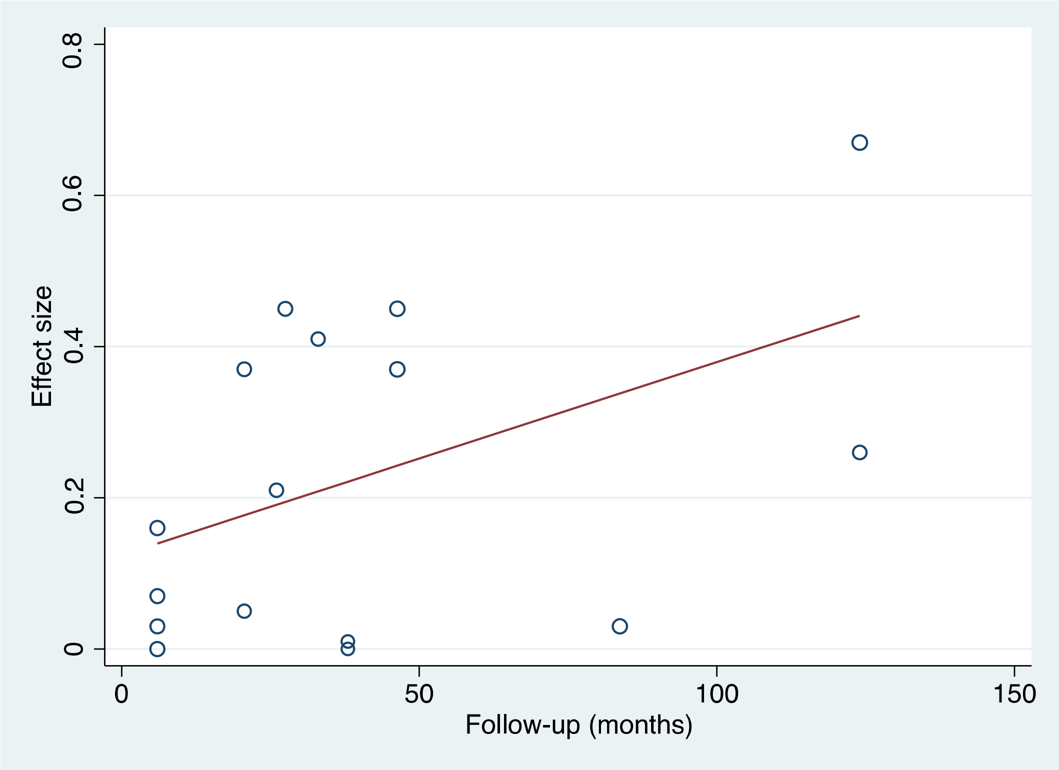

Supplementary Figure S3. Association between correlations on recurrence according to preoperatory factors and age (years).

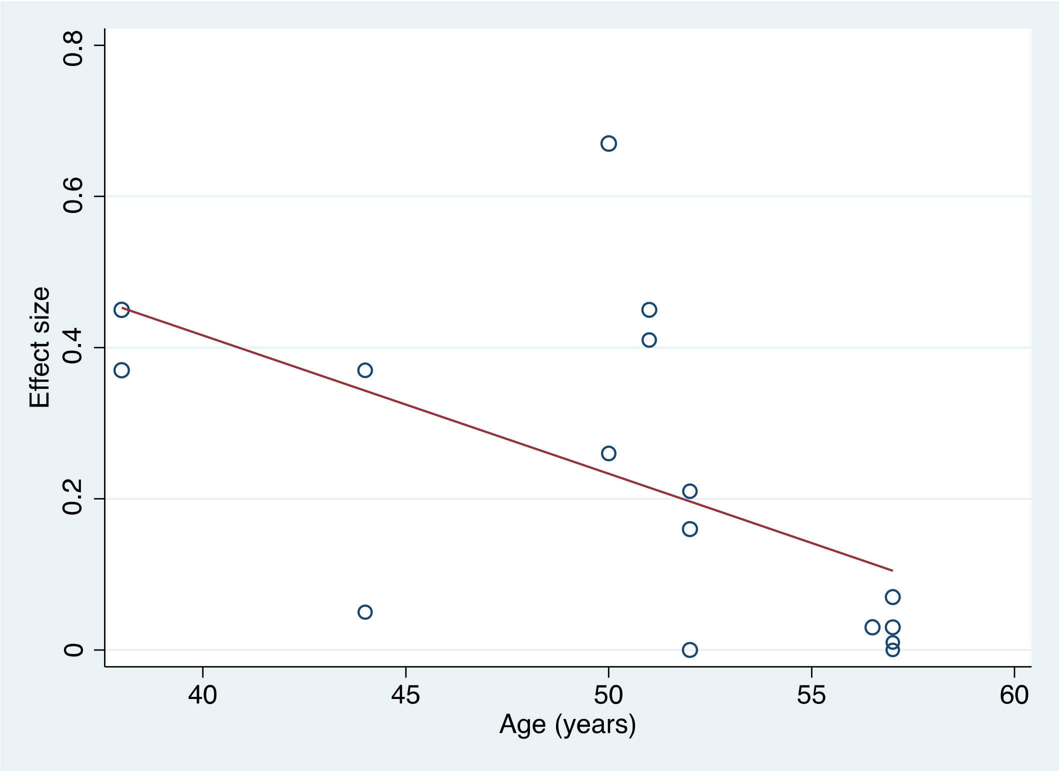

Supplementary Figure S4. Association between correlations on recurrence according to postoperatory factors and follow-up (months).

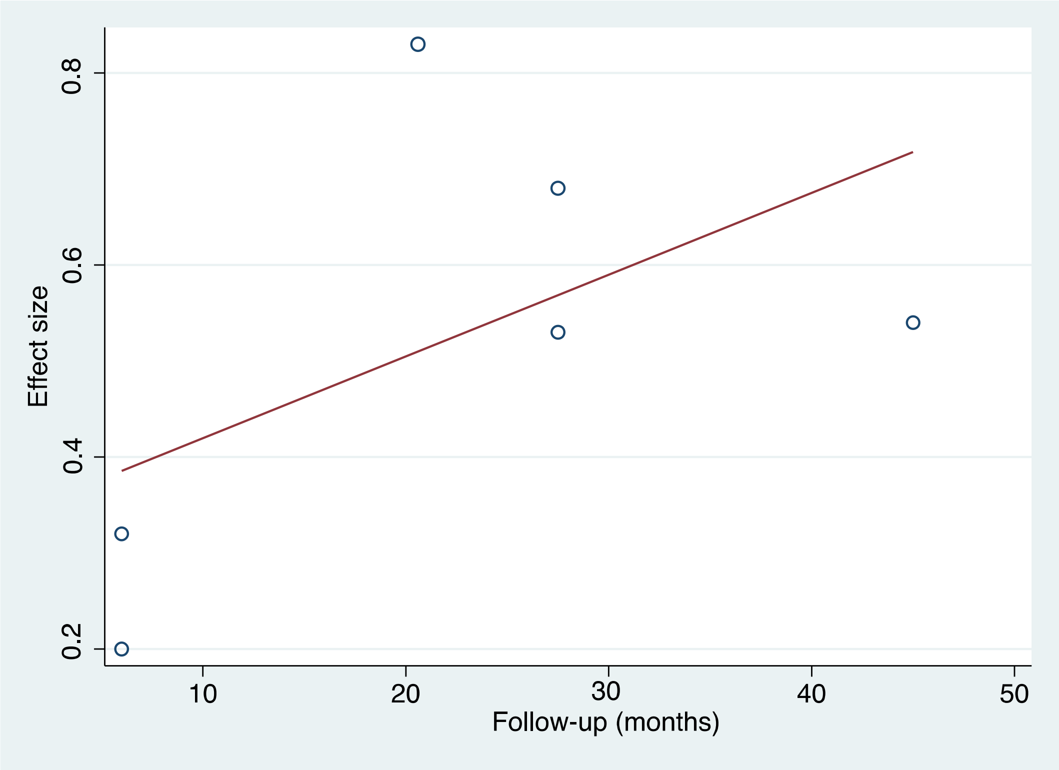

Supplementary Figure S5. Association between correlations on recurrence according to postoperatory factors and age (years).

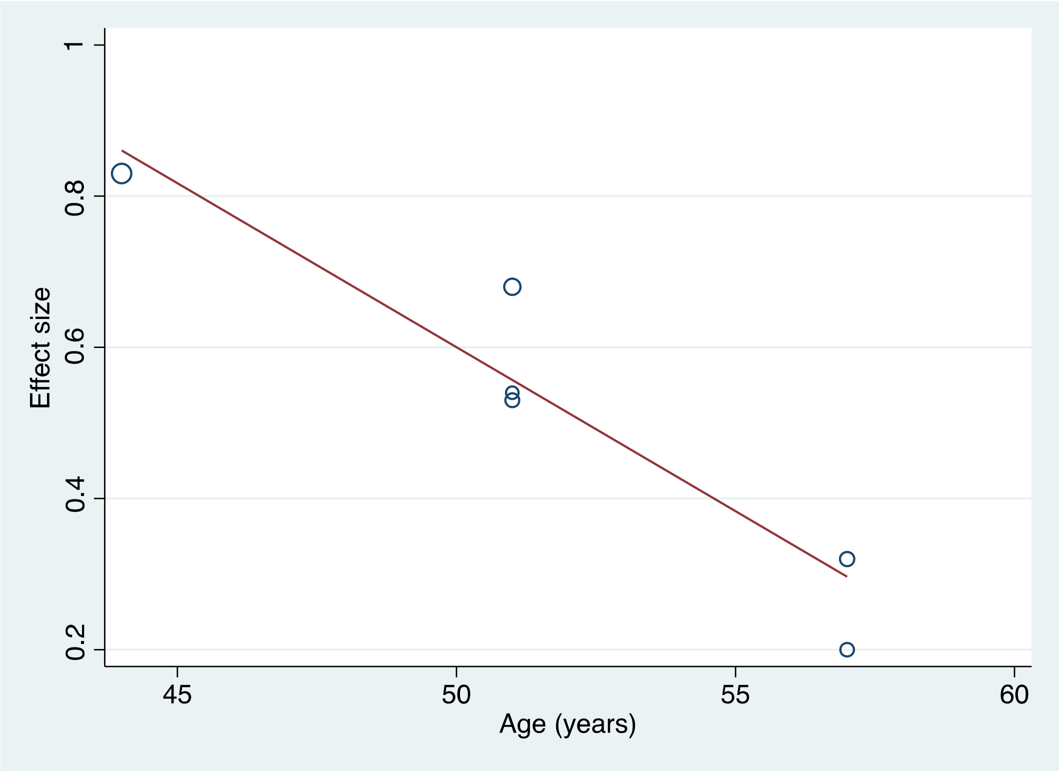

Supplement: Supplementary file 1 [file jcm-10-05753-s001.zip › jcm-1474096-supplementary.pdf]
